# pH-Responsive Hollow Mesoporous Silica Nanoparticles with Fludarabine for Cancer Therapy

**DOI:** 10.3390/nano15171308

**Published:** 2025-08-25

**Authors:** Sung Soo Park, Chang-Sik Ha

**Affiliations:** 1 Department of Polymer Nano Engineering, Dong-Eui University, Busan 47340, Republic of Korea; 2 Department of Polymer Science and Engineering, Pusan National University, Busan 46241, Republic of Korea; csha@pusan.ac.kr

**Keywords:** hollow mesoporous silica, alkylammonium, fludarabine, drug delivery, cancer therapy

## Abstract

In this work, alkylammonium-functionalized hollow mesoporous silica as a nonocarrier of drugs was synthesized to realize enhanced cancer therapy by pH stimuli for sustained drug release. First, functionalized hollow mesoporous silica nanoparticles (Hollow MSNs) were synthesized using dodecyl dimethyl(3-sulfopropyl)ammonium hydroxide (DDAPS), sodium dodecyl sulfate (SDS), and triethanolamine as structure-directing agents, while tetraethyl orthosilicate (TEOS) and *N*-trimethoxysilypropyl-*N*,*N*,*N*-trimethylammonium chloride (TMAPS) were used as silica sources under basic condition via the sol–gel process. The structure and morphology of the alkylammonium-functionalized hollow mesoporous silica nanoparticles (Hollow MSN-N^+^CH_3_) were characterized by X-ray diffraction (XRD), scanning electron microscopy (SEM), transmission electron microscopy (TEM), N_2_ adsorption–desorption analysis, and Fourier transform infrared (FT-IR) spectroscopy. The functionalized hollow MSNs had a particle size of about 450 nm and a shell thickness of about 60 nm with uniform size. The nanoparticle had a surface area of 408 m^2^g^−1^, pore volume of 0.8 cm^3^g^−1^, and a uniform pore diameter of 45.9 Å. In the cancer cell viability test with a MCF-7 cell, fludarabine-incorporated and alkylammonium-functionalized hollow mesoporous silica nanoparticles (Flu/Hollow MSN-N^+^CH_3_) showed excellent cancer cell death comparable with pure fludarabine drug with the controlled drug release by pH stimuli. It is suggested that our current materials have potential applicability as pH-responsive nanocarriers in the field of cancer therapy.

## 1. Introduction

Drug delivery technologies have made a significant contribution to the development of many pharmaceutical products that improve patients’ health by improving drug delivery to the target site and delivering the right amount of drug [1]. Currently, targeted therapy, which uses nanoparticles as a delivery system in cancer treatment to ensure site-specific delivery and release of drugs, is receiving much attention and is being actively studied by many researchers [2,3,4].

Meanwhile, controlled drug release along with efficient drug delivery to the target is also very important for maximizing treatment. Injecting high doses of drugs into the human body can cause damage to healthy organs or tissues in humans by causing undesirable interactions between the drugs and normal cells [5,6]. Therefore, many researchers are making great efforts to overcome these disadvantages by using a controlled drug delivery system [7,8]. Stimuli-responsive polymers [9,10], hydrogels, and many types of inorganic nanoparticles [6,11,12] are being actively studied using temperature, light irradiation, pH, redox, etc., as external stimuli for drug release control without any device.

In recent years, among various drug delivery systems, mesoporous silica nanoparticles (MSNs) are receiving a significant amount of attention in the field of controlled drug delivery because of easy synthesis and surface functionalization, tunable pore size, large surface area, and biocompatibility [13,14,15]. The MSNs have silica walls and uniform nanopores, so they can protect drugs or other compounds and prevent leakage with excellent biocompatibility and high loading capacity [6,16]. Also, since MSNs can be easily functionalized on both the inner and outer surfaces, the premature release of drugs or cargo from the pores can be prevented [6,17]. The MSNs were functionalized with various organic moieties such as aliphatic and aromatic groups, including N, O, and S atoms and their derivatives, to use as drug carriers with controlled release properties and toxicity to cancer cells [18,19,20,21,22,23,24,25,26,27]. The functionalized MSNs exhibited the controlled release behavior of drugs in nanopores by various stimuli, such as pH, light, temperature, enzyme, redox, etc. [18,19,20,21,22,23,24,25,26,27].

Meanwhile, hollow mesoporous silica (HMS) nanoparticles have large pores in the core and nanopores in the shell. Therefore, they can be expected to exhibit dual-drug delivery capability, high drug loading capacity, and different drug release behaviors due to their bimodal pore structure. Many researchers have reported studies on controlled drug release and tests of cancer cell death using drug-incorporated HMS. Ma et al. [28] reported a study on the integrated hollow mesoporous silica nanoparticles for target Drug/siRNA co-delivery. The study simultaneously delivered both doxorubicin (Dox) and siRNA against the Bcl-2 protein into the HeLa cells; the expression of the anti-apoptotic protein Bcl-2 was successfully suppressed, leading to an enhanced therapeutic efficacy. Liu et al. [29] reported on the single peptide ligand-functionalized uniform hollow mesoporous silica nanoparticles achieving dual-targeting drug delivery to tumor cells and angiogenic blood vessel cells. The pharmacodynamic study suggested that, compared with their unmodified counterparts, doxorubicin-loaded tHMSN had an enhanced inhibitory effect on MDA-MB-231 cells and HUVECs in vitro. Chakravarty et al. [30] reported on hollow mesoporous silica nanoparticles for tumor vasculature targeting and positron emission tomography (PET) image-guided drug delivery. ^64^Cu-NOTA-HMSN-PEG-cRGDyK exhibited integrin-specific uptake in vitro and in vivo. She et al. [31] reported on the functionalization of hollow mesoporous silica nanoparticles for improved 5-fluorouracil (5-FU) loading. After modification, amine, carboxyl, cyano, and methyl groups were grafted onto the nanoparticles, and the loading capacity of the hollow mesoporous silica nanoparticles for the anticancer drug 5-fluorouracil was precisely controlled through functionalization. Because 5-fluorouracil exhibits greater deprotonation under alkaline conditions, higher drug loading capacities were observed at pH 7.4 and 8.5. Yang et al. [32] on the hollow silica nanocontainers as drug delivery vehicles. Hollow silica nanoparticles (HSNPs) for drug delivery vehicles were synthesized using silica-coated magnetic assemblies, which are composed of many Fe_3_O_4_ nanocrystals, as templates. Doxorubicin, as a model drug, was loaded into the HSNPs, and notable sustained drug release from HSNPs was demonstrated. Xu et al. [33] reported on the facile way of fabricating PEGylated hollow mesoporous silica nanoparticles (HMSN-PEG) and their drug delivery application. The viability of Hep-G2 cells was evaluated using HMSN-PEG loaded with doxorubicin drug molecules. Zhu et al. [34] reported on hollow mesoporous silica nanoparticles with tunable structures for controlled drug delivery. In this study, we reported PEGylated hollow mesoporous silica (HMS-PEG) nanoparticles as drug carriers for drug delivery. Furthermore, we evaluated the in vitro cytotoxicity and cellular uptake of HMS-PEG nanoparticles loaded with DOX drug molecules against HeLa cells and NIH3T3 cells. Liu et al. [35] reported on the redox-responsive hollow mesoporous silica nanoparticles that were constructed via host–guest interactions for controllable drug release. The prepared HMS@β-CD@PPFc system was used to control drug delivery in targeted cancer therapy through redox stimulus. DOX-loaded HMS@β-CD@PPFc was ingested by A549 cells effectively. Furthermore, the redox agent H_2_O_2_ was used to trigger the release of DOX. The cytotoxicity evaluated by the MTT method indicated that HMS@β-CD@PPFc had good biocompatibility and was promising as a drug carrier. Nia et al. [36] reported on the biotemplated hollow mesoporous silica particles as efficient carriers for drug delivery. In this study, the authors synthesized doxorubicin-containing porous silica nanorods, hollow dendritic fiber-like nanostructured silica (DFNS), and ultraporous sponge-like DFNS. They reported that doxorubicin-containing nanorods exhibited superior anticancer effects against breast cancer cells compared to free doxorubicin. Nguyen et al. [37] reported on the enhanced loading and sustained release carrier for doxorubicin delivery using aminated hollow mesoporous silica nanoparticles. The in vivo experiments demonstrated the significant potency of Ce6@THMSNs-based PDT in obliterating primary tumors and inducing persistent tumor-specific immune responses, thus preventing distant metastasis. Recently, Li et al. [38] reported on the modified hollow mesoporous silica nanoparticles as immune adjuvant-nanocarriers for photodynamically enhanced cancer immunotherapy. Polyethylenimine (PEI) hybrid thin-shell hollow mesoporous silica NPs (THMSNs) were applied as adjuvant-nanocarriers and encapsulated with a very small dose of photosensitizer chlorine e6 (Ce6) to realize the synergy of photodynamic therapy (PDT)/immunotherapy.

HMS used in these previous studies was modified with organic moieties such as amine (-NH_2_), carboxyl (-COOH), methyl (-CH_3_), and cyanide (-CN) groups; single peptide ligands; and polymers such as amphiphilic block copolymer (PEG-*b*-PMAFc, PPFc) and polyethyleneimine (PEI). Meanwhile, 5-fluorouracil (5-FU), paclitaxel, and doxorubicin (DOX) were used as model drugs.

Recently, Rani et al. [39] reviewed various previous studies on mesoporous silica nanoparticle-mediated drug delivery aimed at breast cancer treatment. Bobrin et al. [40] reported the therapeutic delivery of polymeric tadpole nanostructures with high selectivity to triple negative breast cancer cells.

Cancer remains a challenge and the leading cause of death for humans. Many studies using MSN as a drug carrier report results on the controlled release of cancer drugs and toxicity studies on cancer cells [4,23,41,42,43,44,45]. In those studies, doxorubicin (DOX), 5-FU, paclitaxel (PTX), camptothecin (CPT), and epirubicin were mainly used as model drugs. Fludarabine phosphate is a synthetic purine nucleoside that inhibits DNA polymerase and is used for the treatment of chronic lymphocytic leukemia. On the other hand, to the best of our knowledge, few studies have been conducted on the controlled drug delivery systems with the alkylammonium-functionalized hollow mesoporous silica using fludarabine phosphate as a model drug for cancer therapy.

In this context, alkylammonium-functionalized hollow mesoporous silica as a nanocarrier of drugs was synthesized to realize enhanced cancer therapy by pH stimuli for sustained drug release in this work. Fludarabine phosphate was used as a model drug for cytotoxicity testing on breast cancer cell MCF-7. The controlled release of fludarabine phosphate from alkylammonium-functionalized hollow mesoporous silica utilizes electrostatic interactions between fludarabine phosphate molecules and alkylammonium groups.

## 2. Materials and Methods

### 2.1. Materials

The following reagents were used for this work: Dodecyl dimethyl(3-sulfopropyl)ammonium hydroxide (DDAPS, TCI (Tokyo, Japan), ≥98.0%), sodium dodecyl sulfate (SDS, Sigma-Aldrich (St. Louis, MO, USA), ≥99.0%), tetraethyl orthosilicate (TEOS, Sigma-Aldrich, 98%), *N*-trimethoxysilypropyl-*N*,*N*,*N*-trimethylammonium chloride (TMAPS, Gelest (Morrisville, PA, USA), 50% in methanol), triethanolamine (Sigma-Aldrich, 98%), ammonia aqueous solution (Sigma-Aldrich, 25% in H_2_O), ethanol (anhydrous) (Junsei Chemical (Tokyo, Japan), 99%), hydrochloric acid (Matsunoen Chemicals Ltd. (Osaka, Japan), 35% in H_2_O), and fludarabine (Sigma-Aldrich). All the reagents were used without further purification.

### 2.2. Synthesis of Alkylammonium-Functionalized Hollow Mesoporous Silica

The alkylammonium-functionalized hollow mesoporous silica was synthesized as reported in our previous work [46].

**Scheme 1:**
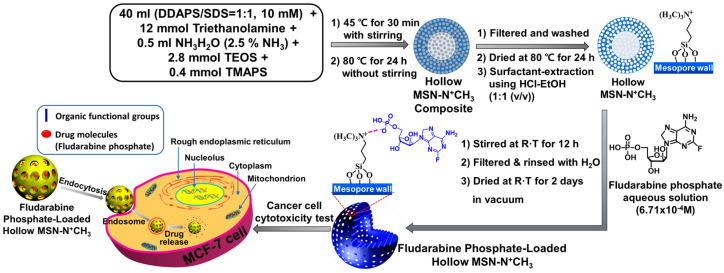
An illustration for the preparation of Hollow MSN-N^+^CH_3_ and the cancer cell cytotoxicity test.

A total of 40 mL of an aqueous surfactant mixture was prepared with DDAPS/SDS = 1:1 (mol ratio) (10 mM) in water. A total of 1.80 g of triethanolamine (12 mmol) and 1 mL of ammonia aqueous solution (2.5% NH_3_ in H_2_O) were added to the surfactant mixture solution. After stirring at room temperature for 1 h to obtain a homogeneous mixture, 630 μL of TEOS (2.8 mmol) and 224 μL of TMAPS (0.4 mmol) were added and heated at 45 °C for 30 min with stirring. The total mixture was heated at 80 °C for another 24 h under static condition. The obtained product was isolated, washed with water and ethanol several times by filtration, and dried in an oven at 80 °C for 24 h. The resulting product was designated as ‘Hollow MSN-N^+^CH_3_ composite’. After this procedure, solvent extraction was conducted to remove the surfactant from the mesopores. Hollow MSN-N^+^CH_3_ composite was stirred in 150 mL of EtOH containing 3 mL of 35 wt% HCl aqueous solution at 60 °C for 12 h, and filtered and washed with EtOH. This process was repeated two times. The product was dried at 80 °C for 24 h. The sample was designated as ‘Hollow MSN-N^+^CH_3_’. The synthesis process is concicely shown in Scheme 1.

### 2.3. Synthesis of Alkylammonium-Functionalized and Fludarabine-Loaded Hollow Mesoporous Silica

A total of 0.6 g of Hollow MSN-N^+^CH_3_ was suspended in 30 mL of 6.7 × 10^−4^ M-fludarabine (Flu) solution in H_2_O and stirred at room temperature for 12 h. The solid sample was filtered and rinsed several times with H_2_O and then dried at room temperature for 2 days in a vacuum oven (1 torr). The resulting product was designed as ‘Flu/Hollow MSN-N^+^CH_3_’.

### 2.4. Drug Releasing Test

A total of 20 mg of Flu/Hollow MSN-N^+^CH_3_ was added to 5 mL of aqueous solution and the dispersion was filled into a dialysis bag (cutoff molecular weight (Mw) = 1.2 kDa). The bag with dispersion was immersed in 20 mL of aqueous solution of different pH (4.8, 7.4, 9.0) at 37 °C in a shaking incubator. At the specific time intervals, 2 mL of external medium was taken and replaced with the same volume of new aqueous solution immediately. The amount of released fludarabine (Flu) ($\%R_{t})$ was calculated by measuring intensity by a UV–vis spectrophotometer at 263 nm.

(1) $$\%R_{t}=\frac{C_{t}\cdot V_{1}+V_{2}\cdot (C_{t-1}+C_{t-2}+\ldots +C_{0})}{W_{0\;}\cdot L}\times 100\%$$

The above equation was applied to calculate the drug release degree of the samples. C*_t_* is the drug concentration at time interval *t*; C*_t_*_−1_ + C*_t_*_−2_ are drug concentrations prior to time interval *t* (C_0_ = 0); V_1_ is the total volume of the release bath (25 mL); and V_2_ is the volume extracted for UV–vis analysis (2 mL). W_0_ is the initial weight of the Flu/Hollow MSN-N^+^CH_3_ and L is the drug loading capacity of the Flu/Hollow MSN-N^+^CH_3_ [47]. Also, calibration curves of fludarabine in aqueous solution at different pH (pH 4.8, 7.4, 9.0) were used to translate UV–vis intensity to the concentration of fludarabine (Flu) in the medium.

### 2.5. Cell Culture

Human breast adenocarcinoma (MCF-7) cell lines were maintained in Dulbecco’s Modified Eagle’s Medium (DMEM, Welgene, Gyeongsan-si, Republic of Korea), supplemented with 10% fetal bovine serum (FBS, Welgene, Gyeongsan-si, Republic of Korea) and 1% penicillinestreptomycin (Welgene, Gyeongsan-si, Republic of Korea) at 37 °C with 5% CO_2_ in a 95% humidified atmosphere. Dulbecco’s phosphate-buffered saline (DPBS) was purchased from Welgene (Gyeongsan-si, Republic of Korea). For the cell viability tests, 3-(4,5-dimethylthiazol-2-yl)-2,5-diphenyltetrazolium bromide (MTT) was purchased from Sigma-Aldrich (St. Louis, MO, USA).

### 2.6. Measurements and Characterization

X-ray powder diffraction (XRD, Rigaku Miniflex, Rigaku, Akishima-shi, Japan) was performed using Cu-Kα radiation (λ = 1.5418 Å) at 30 kV and 15 mA. The characteristic surface functional groups were analyzed by Fourier transform infrared spectroscopy (FTIR, FTIR-4100, JASCO, Chiba-shi, Japan) at a scanning range of 400–4000 cm^−1^ in KBr. Transmission electron microscopy (TEM, TALOS F200X, Thermo Fisher Scientific, Waltham, MA, USA) was performed at an accelerating voltage of 200 kV. High resolution low voltage scanning electron microscopy (HRLV-SEM, JSM-7900F, JEOL, Akishima-shi, Japan) was conducted at an operating voltage of 2.0 kV. The adsorption/desorption isotherms of nitrogen at 77 K were measured using a Nova 4000e (Quantachrome, Boynton Beach, FL, USA) surface area and pore size analyzer. Before starting the measurements, each sample was outgassed for 12 h under vacuum at 343 K. The pore surface area was calculated by Brunauer–Emmett–Teller (BET) method and the pore size distribution curve was obtained from an analysis of the adsorption branch using the Barrett–Joyner–Halenda (BJH) method. Ultraviolet–visible (UV–vis) spectrophotometry (Hitachi U-2010, Hitachi, Hitachi-shi, Japan) was used to measure the drug release rate.

## 3. Results

### 3.1. Synthesis of Hollow MSN-N^+^CH_3_ and Flu/Hollow MSN-N^+^CH_3_

Scheme 1 presents a schematic illustration of the synthesis of Hollow MSN-N^+^CH_3_ and Flu/Hollow MSN-N^+^CH_3_. First, the alkylammonium-functionalized hollow mesoporous silica was synthesized using dodecyl dimethyl(3-sulfopropyl)ammonium hydroxide (DDAPS), sodium dodecyl sulfate (SDS), and triethanolamine as structure-directing agents, and tetraethyl orthosilicate (TEOS) and *N*-trimethoxysilypropyl-*N*,*N*,*N*-trimethylammonium chloride (TMAPS) as silica sources under basic condition via the self-assembly and sol–gel reaction process by the one-pot synthesis method. Drug molecules (Flu) were introduced on the surface of the pores by the electrostatic interaction between alkylammonium groups and drug molecules.

### 3.2. Characterization of Hollow MSN, Hollow MSN-N^+^CH_3_, and Flu/Hollow MSN-N^+^CH_3_

#### 3.2.1. Scanning Electron Microscopy (SEM) and Transmission Electron Microscopy (TEM)

**Figure 1:**
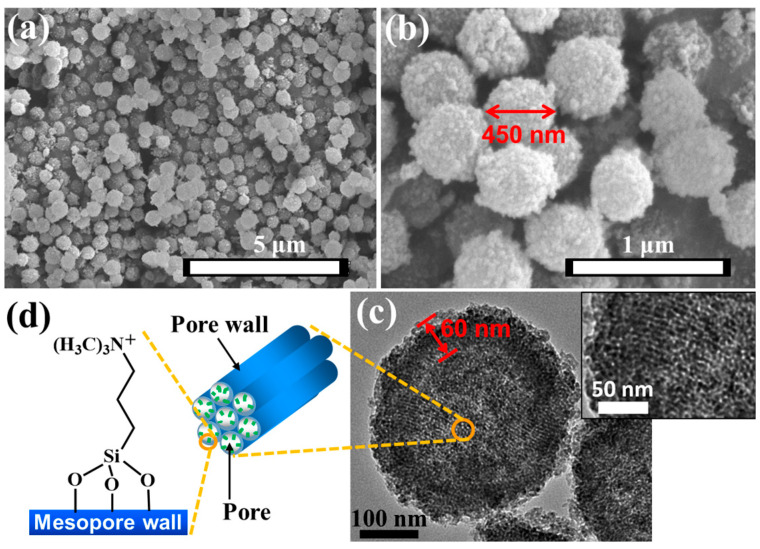
SEM and TEM images (**a**,**b**) of Hollow MSN-N^+^CH_3_ (**c**). A schematic diagram of the alkylammonium-functionalized mesopore wall (**d**). The inset in (**c**) shows a high-magnified TEM image.

Figure 1 shows SEM (a,b) and TEM images (c) of Hollow MSN-N^+^CH_3_. The alkylammonium-functionalized hollow mesoporous silica (Hollow MSN-N^+^CH_3_) has a particle size of about 450 nm and a shell thickness of about 60 nm with uniform size as shown in TEM image (Figure 1c). The surfaces of the particles show rather rough surfaces with smaller particles. The TEM image showed that the mesopores in the shell have an ordered 2D hexagonal mesostructure with parallel strips and uniform pore size [48,49,50], even after the surface of the mesopores was functionalized with alkylammonium groups. (Figure 1d).

#### 3.2.2. XRD Patterns

**Figure 2:**
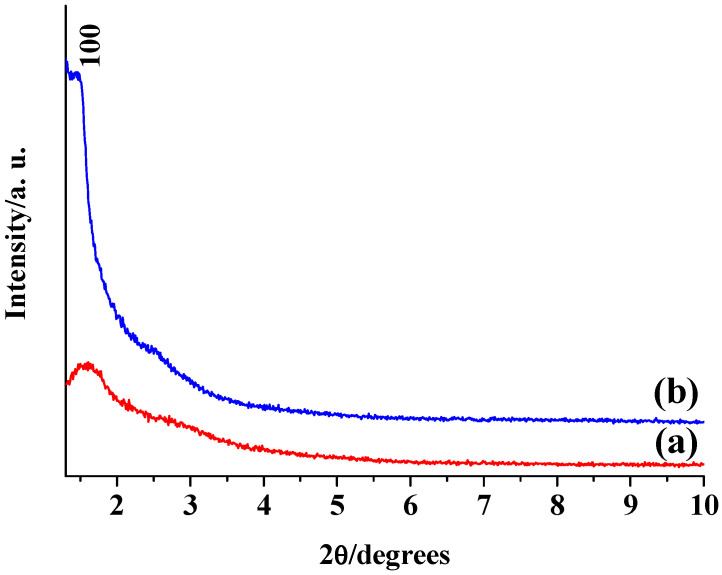
XRD patterns of (**a**) Hollow MSN and (**b**) Hollow MSN-N^+^CH_3_.

Figure 2 shows XRD patterns of (a) Hollow MSN and (b) Hollow MSN-N^+^CH_3_. The alkylammonium-functionalized hollow mesoporous silica (Hollow MSN-N^+^CH_3_) shows one peak assigned by (100) reflection at 2θ = 1.44° (Figure 2b). The (110), (200), and (210) reflections in the range of 2θ = 2.5~6° seen on the 2D-hexagonal mesostructure are not clear due to the broad XRD pattern. The result indicates that the mesopores have an ordered structure in a small range [48,49,50]. However, it can be confirmed from the TEM image shown in Figure 1c that Hollow MSN-N^+^CH_3_ has uniformly sized mesopores despite less ordering. Here, to compare the usefulness of the alkylammonium groups in Hollow MSN-N^+^CH_3_, hollow mesoporous silica nanoparticles (Hollow MSN) without organic functional groups were prepared by calcination of Hollow MSN-N^+^CH_3_ at 550 °C for 4 h in air. Hollow MSN showed lower intensity and broader peaks compared to Hollow MSN-N^+^CH_3_ (Figure 2a). The result may be due to the disappearance of organic groups present in the pore walls and the collapse of the mesostructure due to the shrinkage of the pore walls by treatment at high temperatures of 550 °C [51]. The 2θ values of Hollow MSN increased to 1.61°. The result occurs by the shrinkage of the mesopore walls at high temperatures [51].

#### 3.2.3. N_2_ Adsorption–Desorption Isotherms and Pore Size Distributions

**Figure 3:**
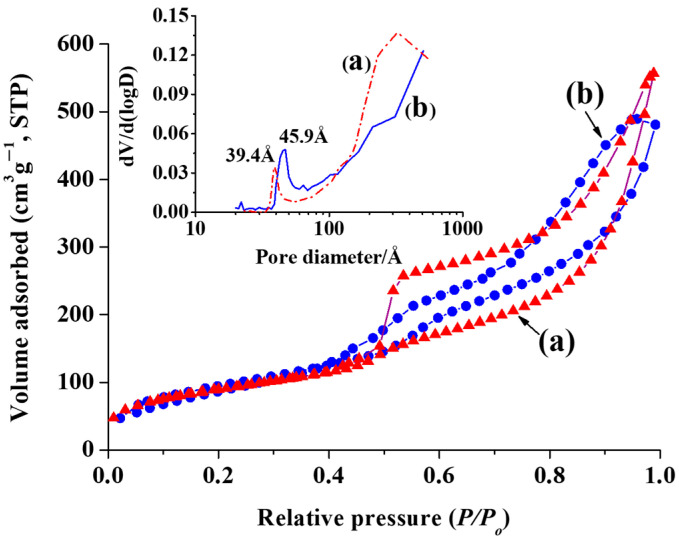
N_2_ adsorption–desorption isotherms of (**a**) Hollow MSN and (**b**) Hollow MSN-N^+^CH_3_. The inset shows pore size distributions of (**a**) Hollow MSN and (**b**) Hollow MSN-N^+^CH_3_.

Figure 3 shows N_2_ adsorption–desorption isotherms of (a) Hollow MSN and (b) Hollow MSN-N^+^CH_3_. The inset shows pore size distributions of (a) Hollow MSN and (b) Hollow MSN-N^+^CH_3_. Hollow MSN-N^+^CH_3_ has a BET surface area of 405 m^2^g^−1^ and a pore volume of 0.80 cm^3^g^−1^. The pore diameter of Hollow MSN-N^+^CH_3_ was 45.9 Å. The result agrees well with the TEM image in Figure 1c. Also, the pore size distribution gradually increased to the right of the plot. The result is attributed to large pores in the core of hollow nanoparticles and voids formed by the aggregation of nanoparticles [18,46]. The pore diameter of Hollow MSN treated at high temperature was 39.4 Å, which is smaller than that of Hollow MSN-N^+^CH_3_. It is due to the shrinkage of the mesopores [51]. Also, as in the case of Hollow MSN-N^+^CH_3_, a broad pore size distribution was shown around 300 Å. BET surface area and pore volume of Hollow MSN were 358 m^2^g^−1^ and 0.90 cm^3^g^−1^, respectively.

#### 3.2.4. FT-IR Spectra

**Figure 4:**
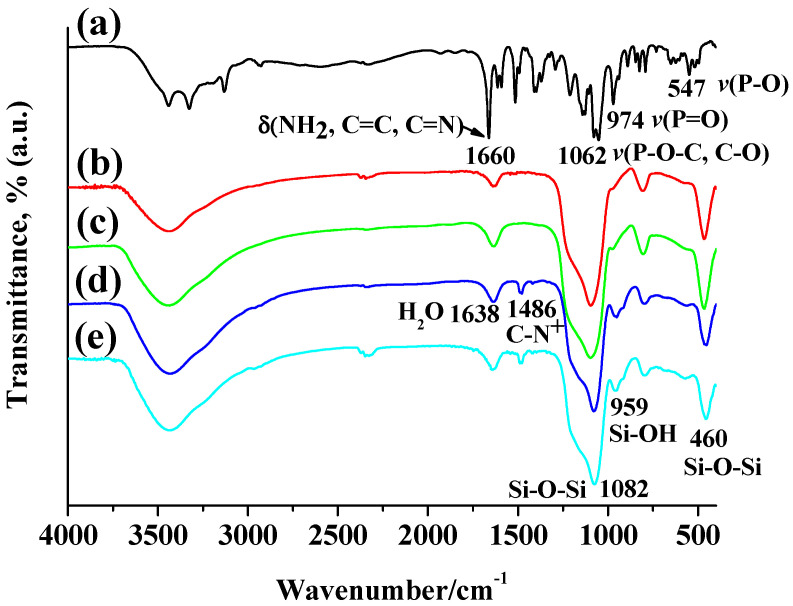
FT-IR spectra of (**a**) fludarabine phosphate, (**b**) Hollow MSN, (**c**) Flu/Hollow MSN, (**d**) Hollow MSN-N^+^CH_3_, and (**e**) Flu/Hollow MSN-N^+^CH_3_.

Figure 4 shows FT-IR spectra of (a) fludarabine phosphate, (b) Hollow MSN, (c) Flu/Hollow MSN, (d) Hollow MSN-N^+^CH_3_, and (e) Flu/Hollow MSN-N^+^CH_3_. The alkylammonium-functionalized hollow mesoporous silica (Hollow MSN-N^+^CH_3_) exhibited a characteristic peak at 1486 cm^−1^ due to the C-N bond in alkylammonium moieties in FT-IR spectrum (Figure 4d) [18,52]. The characteristic peaks due to the silica framework exhibited at 460 and 1082 cm^−1^ for Si-O-Si, 959 cm^−1^ for Si-OH [6,18,52]. Also, Hollow MSN treated at a high temperature of 550 °C showed peaks due to the silica framework in a position similar to that of Hollow MSN-N^+^CH_3_ (Figure 4b). On the other hand, the peak caused by the C-N bond did not appear. And, as high temperature treatment causes dehydration of Si-OH groups in the silica framework, Si-OH groups are reduced by condensation to form Si-O-Si bonds [48,53]. Therefore, as shown in Figure 4b, the intensity of the FT-IR peak for Si-OH was significantly reduced. Figure 4c shows the FT-IR spectrum of fludarabine-incorporated Hollow MSN (Flu/Hollow MSN) by drug solution treatment. The characteristic peaks for fludarabine in the spectrum were not observed. The result may be attributed to the low affinity between silanol groups (Si-OH) present on the surface of mesopores and fludarabine molecules containing negatively charged phosphate groups [54]. Most of the fludarabine molecules were lost during the introduction of the fludarabine molecules into the hollow MSN, including the sample rinsing process. For the FT-IR spectrum of fludarabine-introduced Flu/Hollow MSN-N^+^CH_3_ (Figure 4e), the characteristic peak of fludarabine was not clearly observed. It is, however, the result of overlapping peak positions of fludarabine and Hollow MSN-N^+^CH_3_.

#### 3.2.5. Incorporation of Fludarabine in Flu/Hollow MSN-N^+^CH_3_

**Figure 5:**
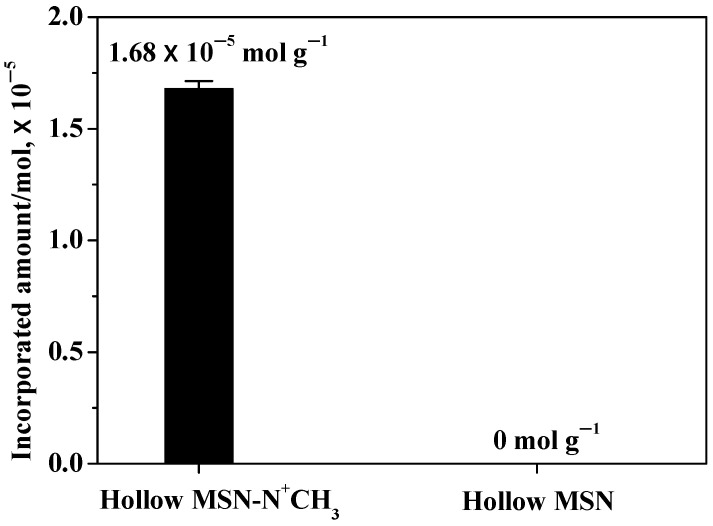
Incorporated amount of fludarabine in Hollow MSN and Flu/Hollow MSN-N^+^CH_3_.

When incorporating fludarabine into Hollow MSN-N^+^CH_3_ and Hollow MSN, the UV–vis technique was used to clearly identify them. The incorporated amount of fludarabine was calculated based on the UV–vis adsorption of the fludarabine solution before and after adsorption to the adsorbent. The alkylammonium-functionalized hollow mesoporous silica (Hollow MSN-N^+^CH_3_) had an adsorption capacity of 1.68 × 10^−5^ mol Flu/g absorbent. This value indicates that 50.1% of the fludarabine contained in the fludarabine mother liquor was incorporated into Hollow MSN-N^+^CH_3_. We used a fludarabine solution at a high concentration to achieve sufficient incorporation. On the other hand, the Hollow MSN, which does not have organic functional groups, hardly adsorbs fludarabine (Figure 5). The result agrees well with the result of the FT-IR spectrum shown in Figure 4c.

#### 3.2.6. Time-Dependent Release Profiles of Fludarabine

**Figure 6:**
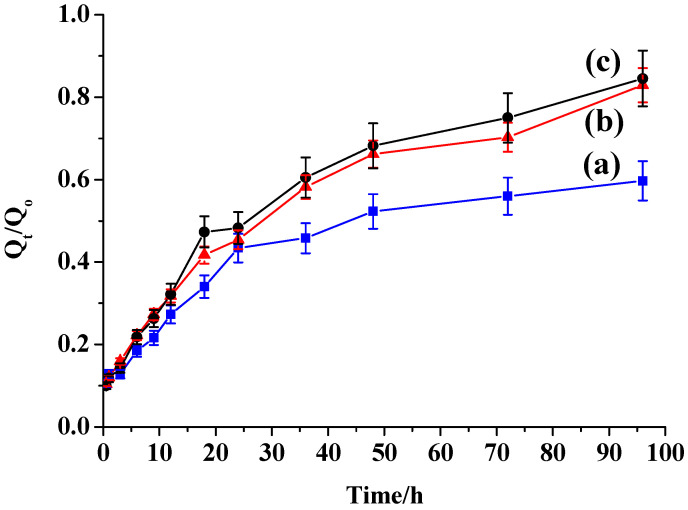
Time-dependent release profiles of fludarabine from Flu/Hollow MSN-N^+^CH_3_, with different pH environments of (**a**) pH 4.8, (**b**) 7.4, and (**c**) 9.0 at 37 °C.

Figure 6 shows time-dependent release profiles of fludarabine from Flu/Hollow MSN-N^+^CH_3_, with different pH environments of (a) pH 4.8, (b) 7.4, and (c) 9.0 at 37 °C. As the release time increases, the released amount of fludarabine (Flu) contained in the Hollow MSN-N^+^CH_3_ increases, and as the contained Flu is depleted, an equilibrium is reached. These results show similar results to previously reported studies on drug-loaded hollow mesoporous silica or non-hollow mesoporous silica materials [6,28,29,32,33,34,35,43,48]. With the pH increase to pH 9.0, the interaction between fludarabine and the alkylammonium group would be hindered as the OH^-^ counter ion in solution interacts with the alkylammonium groups on the mesopore surface. Thereby, the drug release amount increased at a higher pH than pH 4.8.

In this study, approximately 50% of the drug was released from the nanocarrier (Fu/Hollow MSN-N^+^CH_3_) within 30 h. As the release time increased to 96 h under various pH conditions, the release amount of the remaining drug showed a more distinct difference with gradual and continuous release.

Cancer cells, including breast cancer cells, exhibit a lower extracellular pH (pHe) gradient of ~6.7–7.1 and a higher intracellular (pHi) of 7.4, while normal cells have a higher pHe (7.4) and a lower pHi (7.2) [55,56]. Therefore, some studies showed that treating cancer cells with alkaline compounds can effectively treat cancer [57,58]. If the drug-containing nanoparticle enters the inside of the cancer cell through phagocytosis and the drug is easily released at pH 7.4, it will be effective in eradicating the cancer cell.

In this context, the release behavior of the drug in the Flu/Hollow MSN-N^+^CH_3_ was investigated not only under acidic condition but also under basic conditions of pH = 9.0.

#### 3.2.7. Cytotoxicity Test Against Cancer Cells

In this study, we focused on demonstrating that the Flu/Hollow MSN-N^+^CH_3_ synthesized had a significant toxic effect on MCF-7 cells, a cancer cell. In the cancer cell viability test with MCF-7 cells, although fludarabine-incorporated hollow mesoporous silica nanoparticles do not show much better performance than pure fludarabine, the fludarabine-incorporated hollow mesoporous silica nanoparticles show excellent cancer cell death comparable to pure fludarabine. One important point can be considered: When a pure drug is ingested into the body, only a small amount of the drug molecule acts on cancer cells. And the rest of the drugs will adversely affect normal cells. To solve this problem, many researchers studied controlled drug release systems. In this context, the controlled fludarabine release in this work is of great significance. MCF-7 showed cell viability of 2.5% when 100 μg Flu/Hollow MSN-N^+^CH_3_ per 1 mL culture medium was dosed. Meanwhile, in this study, the cytotoxicity against MCF-7 cells was studied using free fludarabine and the Flu/MSN-N^+^CH_3_ containing fludarabine with the amount 0~100 μg Flu/Hollow MSN-N^+^CH_3_ per 1 mL culture medium, respectively. As the amount of Flu/MSN-N^+^CH_3_ increases from 0 to 100 μg Flu/Hollow MSN-N^+^CH_3_ per 1 mL culture medium, the amount of fludarabine in this sample increases from 0 to 0.62 μg Flu/Hollow MSN-N^+^CH_3_ per 1 mL culture medium. In other words, the amount of fludarabine contained in the Flu/MSN-N^+^CH_3_ is approximately 1/163 times the amount of free fludarabine used. Nevertheless, it showed notable cytotoxicity against MCF-7 cells.

**Figure 7:**
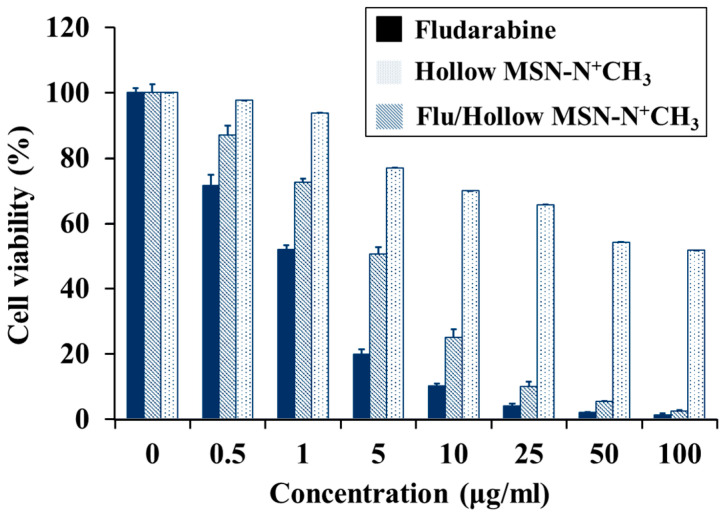
In vitro cytotoxicity of Flu/Hollow MSN-N^+^CH_3_ against MCF-7 cancer cells.

Therefore, the Flu/Hollow MSN-N^+^CH_3_ synthesized in this study can be considered to be a meaningful active substance for MCF-7 cells (Figure 7).

Meanwhile, interestingly, the Hollow MSN-N^+^CH_3_ without Flu shows increased cytotoxicity as the dosage amount increases. In previous research, Tao et al. [59] reported on mesoporosity and functional group-dependent endocytosis and cytotoxicity of silica nanomaterials. In the study, they carried out the cytotoxicity test against the human T-cell lymphoma (Jurkat) and human neuroblastoma cells (SK-N-H) using quaternary ammonium-functionalized mesoporous silicas (SBA-N, MCM-N, and SMS-N). SBA-N, MCM-N, and SMS-N samples showed different cytotoxicity depending on dosage time and cell type. However, the cause of cytotoxicity was not explained in detail.

When the concentration of the Hollow MSN-N^+^CH_3_ was as low as 0.5 μg/mL and 1 μg/mL, cell viability was 98% and 94%, respectively. Meanwhile, the drug-containing Hollow MSN-N^+^CH_3_ (Fu/Hollow MSN-N^+^CH_3_) exhibited a cell viability of 86% with the concentration of 0.5 μg Flu/Hollow MSN-N^+^CH_3_ per 1mL culture medium and a cell viability of 72% with 1 μg Flu/Hollow MSN-N^+^CH_3_ per 1mL culture medium, respectively. This study showed cytotoxicity results for various doses of Fu/Hollow MSN-N^+^CH_3_ over a wide range. However, it is believed that the applicability will increase if the adjusted Fu/Hollow MSN-N^+^CH_3_ concentrations are used. To support the desirable cytotoxicity of the Flu/Hollow MSN-N^+^CH_3_ for future cancer therapy application, further work will be done on their cytotoxicity in vivo as well as their cytotoxicity using more breast cancer cell lines, normal cell lines (e.g., MRC5), and so on.

## 4. Conclusions

In this work, the alkylammonium-functionalized hollow mesoporous silica (Hollow MSN-N^+^CH_3_) nanoparticles were synthesized using zwitterionic surfactant (*N*-dodecyl-*N*,*N*-dimethyl-3-ammonio-1-propane sulfonate, DDAPS), anionic surfactant (sodium dodecyl sulfate, SDS), and triethanolamine as structure-directing agents and TEOS and alkylammonium alkoxysilane (TMAPS) as silica sources. The Hollow MSN-N^+^CH_3_ had a particle size of ca. 450 nm with a shell thickness of ca. 60 nm. The surface area and pore volume of the Hollow MSN-N^+^CH_3_ were 408 m^2^g^−1^ and 0.8 cm^3^g^−1^, respectively, with a uniform pore diameter of 45.9 Å. The Flu/Hollow MSN-N^+^CH_3_ containing fludarabine molecules as a model drug exhibited the controlled release behavior of drug molecules at different pH conditions (pH 4.8, 7.4, 9.0). In the cell cytotoxicity test against MCF-7 cancer cell, fludarabine-incorporated and alkylammonium-functionalized hollow mesoporous silica nanoparticles (Flu/Hollow MSN-N^+^CH_3_) showed the cell viability of 2.5%. The result implies that the Hollow MSN-N^+^CH_3_ possesses a promising cancer-cell-killing ability, comparable to the pure fludarabine drug. It is considered that our current materials have excellent applicability as pH-responsive nanocarriers in the field of medical treatment.

## Data Availability

The original contributions presented in this study are included in the article. Further inquiries can be directed to the corresponding author.
